# Chinese as a second language anxiety and intervention in a technology-assisted environment: a mini-review

**DOI:** 10.3389/fpsyg.2026.1801765

**Published:** 2026-05-11

**Authors:** Bo Yu

**Affiliations:** International College of Chinese Language and Culture, Chongqing Normal University, Chongqing, China

**Keywords:** Chinese as a second language (CSL), intervention strategies, language learning anxiety, online learning, technology-assisted language learning

## Abstract

This mini-review examines research on anxiety among learners of Chinese as a second language (CSL) in technology-assisted environments. It synthesizes findings from empirical studies published between 2015 and 2025, focusing on the unique manifestations of anxiety in digital settings, key influencing factors (e.g., technological features, individual differences, instructional design), and evidence-based intervention strategies. Results indicate that technology can both alleviate and exacerbate anxiety: tools such as VR and gamification show promise in reducing oral and communicative anxiety, whereas technical challenges and emotional isolation may heighten distress. Notably, the same platform (e.g., video-conferencing) can produce opposite effects depending on task design and learner proficiency. The review also highlights methodological limitations, including small sample sizes and short-term designs, and proposes a Technology-Affect Interaction Framework to guide future research. The review recommends longitudinal, mixed-methods approaches and learner-centered technological ecosystems that systematically support emotional as well as cognitive dimensions of CSL learning.

## 1 Introduction

### 1.1 Background: the rise of technology in CSL and affective considerations

In the 21st century, where globalization and digitalization are deeply integrated, technology-assisted language learning (Computer-Assisted Language Learning, CALL), especially mobile-assisted language learning (Mobile-Assisted Language Learning, MALL), has become an irreversible trend in the field of language education (Kukulska-Hulme and Viberg, 2018). The widespread use of mobile devices such as smartphones and tablets, as well as the empowerment of technologies like artificial intelligence and big data, have greatly expanded the temporal and spatial boundaries of language learning, providing learners with an unprecedented personalized and immersive learning experience. At the same time, the vigorous development of International Chinese Language Education has led to an increasingly large group of learners of Chinese as a second language (CSL), and the emotional factors in their learning process, especially language learning anxiety, have also received increasing attention from researchers.

Language learning anxiety refers to a distinctively strong anxiety emotion that learners experience due to the uniqueness of the language learning process. It is a key variable that affects the effectiveness of second language acquisition. For Chinese language learners, the unique writing system of Chinese characters, the complex tone and the profound cultural connotations may cause them to face greater cognitive load and psychological pressure compared to learners of other languages, leading to language anxiety and impacting their performance (Qian, 1999).

### 1.2 Conceptual clarification: defining “anxiety” in this review

In the language learning literature, “anxiety” is a multifaceted construct. For the purpose of this review, the framework established by Horwitz et al. is adopted and adapted, which defines foreign language classroom anxiety (FLCA) as a distinct complex of self-perceptions, beliefs, feelings, and behaviors related to classroom language learning arising from the uniqueness of the language learning process (Horwitz et al., 1986). The focus of this review is specifically on situated, state-level anxiety—the subjective, transient apprehension experienced by learners in technology-assisted CSL learning environments. While acknowledging the influence of more stable trait anxiety (a general personality predisposition to anxiety), the analysis centers on how technological and pedagogical factors evoke or modulate situation-specific anxiety states.

Anxiety is further differentiated based on the language skill domain. The manifestations are examined distinctly in relation to listening, speaking, reading, and writing in digital contexts. A primary aim of this synthesis is to uncover whether and how technology impacts anxiety differently across these skill modalities.

Attention is also given to the theoretical distinction between debilitating anxiety (which impairs performance) and facilitating anxiety (which may motivate heightened effort). The literature reviewed herein primarily reports on anxiety as a negative, performance-interfering emotion. Consequently, this review predominantly addresses anxiety in its debilitating form, while noting that certain technological designs might theoretically transform anxiety into a more facilitative experience—a potential avenue for future inquiry. By adopting these conceptual clarifications, consistency and precision are ensured in interpreting and synthesizing the findings across the diverse studies included in this mini-review.

### 1.3 Aims and scope of the present mini-review

This study aims to conduct a mini-review of the research on Chinese second language anxiety in a technology-assisted environment. The reason for adopting this mini-review format is that although there have been many empirical explorations in this field, the research conclusions are often scattered or even contradictory, and there is a lack of systematic integration and critical analysis. A comprehensive systematic review is undoubtedly ideal, but given that the current research volume is still in the accumulation stage and the quality varies, a mini-review that focuses on core issues, aims to outline the key context and highlight contradictions, can provide timely and concentrated insights for clarifying the current research status, identifying methodological flaws, and guiding future directions.

This mini-review will be conducted around the following three core questions:

1. In a technology-assisted environment, what are the unique manifestations of Chinese second language learners' anxiety? What are the main influencing factors (such as technological characteristics, teaching design, individual differences)? The technology environment not only may change the form of traditional classroom anxiety, but its interaction with the learning difficulties of Chinese itself is more likely to generate unique anxiety experiences, which urgently need to be deeply analyzed.
2. What technology intervention strategies have been used in existing research to alleviate Chinese second language anxiety? What are the empirical effects of these strategies, and what is their mechanism? The effectiveness evidence of current intervention measures (such as using VR/AR to create a safe context, based on AI for immediate feedback, gamification incentives, etc.) is uneven, and their targeted effects on different types of anxiety (such as oral anxiety, writing anxiety) are not clear, and require systematic evaluation.
3. What are the main methodological limitations and theoretical gaps in this field of research? What paths should future research prioritize? A large number of studies have problems such as small sample size, short cycles, and unrigorous experimental design (Burston, 2015), and most have not deeply revealed the mediating mechanism of technology on anxiety. Identifying these limitations and gaps is crucial for enhancing the scientific nature and practical guidance value of future research.

It is worth noting that the interaction between the technological environment and second language anxiety in Chinese presents a highly complex and contradictory nature, which constitutes the core contradiction that this review needs to address. On the one hand, technology is regarded as a potential remedy for anxiety. Technologies such as virtual reality (VR) and augmented reality (AR) can create safe simulated communication scenarios; adaptive learning systems can provide customized content, reducing feelings of frustration. However, on the other hand, technology itself may also become a new source of anxiety. Network connections, software operations, the sense of coldness in human-computer interaction, and the social gaze pressure from online public expression can all trigger or exacerbate anxiety (Stockwell, 2013). This dual-edged nature of technology has been highlighted in recent empirical studies. Research indicates that, compared to traditional face-to-face teaching, the use of CALL and MALL in instruction can lead to a significant increase in learners' anxiety levels (Dong et al., 2022). This finding contrasts with many studies reporting positive effects, profoundly revealing the complexity of technology's involvement in the emotional domain. It warns us to abandon the simplistic assumption that technology will inevitably reduce anxiety and instead calls for more careful and detailed examinations. Moreover, how individual differences of learners (such as cultural background, motivation type, and self-efficacy) interact with the technological environment and jointly influence the anxiety experience is also an indispensable dimension for understanding this phenomenon.

This mini-review will sort out the existing evidence, integrate and analyze the manifestations, causes, interventions, and research quality of Chinese second language anxiety in a technology-assisted environment, aiming to clarify the current contradictions discovered and provide a clear road map for future construction of more rigorous and effective emotion-enhanced technology-based Chinese language learning environments.

## 2 Methodology

In order to ensure that this review can fully reflect the research progress of Chinese second language anxiety and intervention in the technology assisted environment, this review follows clear literature selection criteria.

### 2.1 Literature search strategy and selection criteria

To ensure a transparent and reproducible review process, a systematic literature search was conducted. The search was performed across major academic databases. Chinese-language publications were primarily retrieved from the China National Knowledge Infrastructure (CNKI). English-language publications were sourced from databases including Web of Science Core Collection, ERIC, and PsycINFO.

The search strategy employed a combination of keywords related to three core concepts: (1) the target language (e.g., “Chinese as a second language,” “CSL,” “汉语二语”), (2) the psychological construct (e.g., “anxiety,” “语虑”), and (3) the learning context (e.g., “technology-assisted,” “online,” “CALL,” “MALL,” “计算机辅助,” “线上”). Boolean operators (AND, OR) were used to refine the search. An illustrative search string for English databases was: (“Chinese as a second language” OR “CSL”) AND (anxiety) AND (“online” OR “technology-assisted” OR “CALL” OR “MALL”). The publication timeframe was set from 2015 to 2025 to capture the evolution of research concurrent with the rapid adoption of educational technology. All the retrieved records were imported into the literature management software, and the duplicate documents were removed.

### 2.2 Study screening, selection, and final inclusion

**Figure 1:**
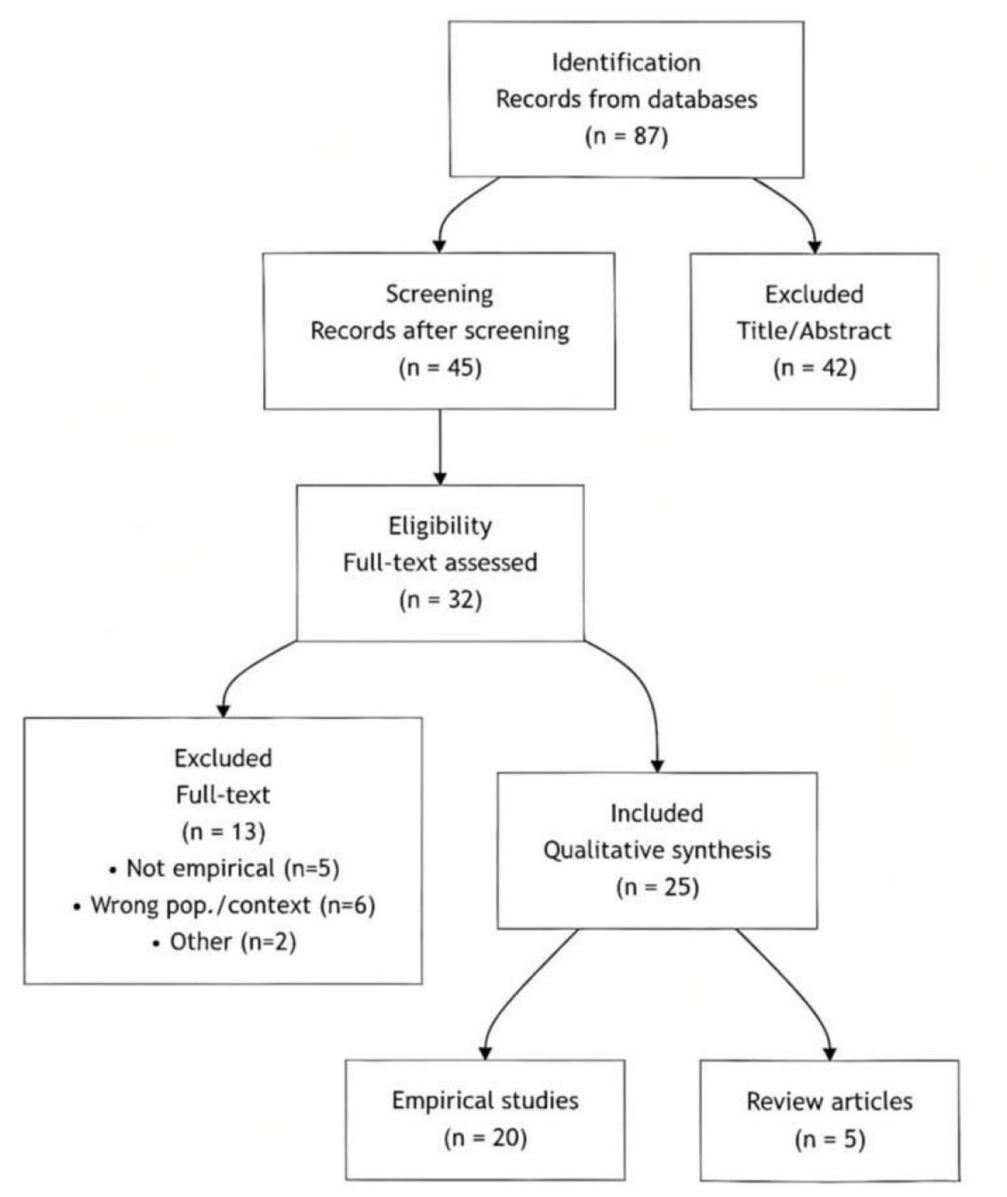
PRISMA flow diagram of the literature search and selection process.

The screening and selection process followed a structured multi-stage protocol, detailed in the PRISMA flow diagram (see Figure 1).

Initial Screening: All retrieved records were compiled, and duplicates were removed. The titles and abstracts of the remaining records were screened against broad relevance criteria. Records were excluded if they were clearly unrelated (e.g., not focusing on CSL learners, or not involving a technology-assisted learning environment).

Full-Text Screening and Eligibility Assessment: The full texts of the remaining records were obtained and rigorously assessed against the following pre-defined eligibility criteria:

Study Type: Empirical research (quantitative, qualitative, or mixed-methods). Theoretical or review articles were excluded but saved for background reading.
Participants: Learners of Chinese as a second or foreign language.
Context: The learning environment must be technology-assisted (e.g., online platforms, mobile applications, virtual reality).
Focus: The study's core variables must include CSL learning anxiety and/or an intervention aimed at it.
Language and Date: Publications in Chinese or English, published between 2015 and 2025.

This process was carried out independently by two researchers, and the screening results were cross checked to ensure the reliability of the decision.

Final Inclusion and Data Extraction: Studies meeting all eligibility criteria were included. For each included study, key data (e.g., authors, technology environment, main findings related to anxiety) were extracted and synthesized. This process resulted in the final corpus of 25 publications for this mini-review.

A Note on Source Types and Rationale: The final corpus comprises 20 empirical studies and 5 review articles. Notably, among the 20 empirical studies, 10 are peer-reviewed journal articles and 10 are Master's theses. This composition aligns with the purpose of a mini-review, which is to provide a timely, focused synthesis and mapping of a developing field rather than an exhaustive systematic review. Including Master's theses allows for a more comprehensive capture of early exploratory work and practitioner-led inquiries. In the synthesis and discussion, we maintain a critical perspective: primary conclusions are grounded in findings from peer-reviewed journal articles, while insights from theses are treated as valuable preliminary evidence and illustrative cases. This treatment also enables us to consider the potential quality differences caused by differences in research methods (such as peer-reviewed research and Dissertations) in the comprehensive analysis.

### 2.3 Overview of review literature

Research on Chinese second language learning anxiety in the past decade has shown the characteristics of multi-dimensional perspective and technology integration. The study found that motivation is negatively correlated with anxiety, while learning strategies positively predict anxiety, and technology tools had complex regulatory effects on anxiety, motivation, strategies and learning outcomes (Lan, 2025). Compared with Chinese and foreign studies, scholars in mainland China mostly use quantitative methods, while international scholars prefer mixed research methods; Gender, language level and cultural background were the significant influencing factors of anxiety (Yao et al., 2022). In addition, the meta-analysis results show that anxiety is negatively correlated with Chinese learning achievement in a small to medium degree, among which beginners are most affected by anxiety, and the proportion of women significantly regulates the relationship between the two (Qiao et al., 2025).

In terms of research on technology assisted Chinese teaching, the research found that mobile applications, Web 2.0 technology, network conference technology and so on are widely used to support Chinese teaching, and mobile applications have become the mainstream research tools in the past decade (Lyu and Qi, 2020). The research also points out that technology application has advantages in improving learning motivation and providing real context, but it also faces challenges such as teachers' and students' lack of technical ability, students' low attendance and retention rate, and difficulties in connecting learning inside and outside the classroom.

To sum up, the existing research has constructed the correlation framework of anxiety, motivation, strategy and technology, but it is necessary to strengthen cross-cultural comparison, long-term tracking and the application of mixed methods to deepen the empirical research of technology intervention anxiety.

### 2.4 Overview of empirical research literature

Twenty empirical studies were identified, including 10 Chinese publications and 10 English publications. These studies mainly explore the influence of variables such as anxiety, motivation, learning strategies and Chinese learning outcomes (such as language skills, academic adaptation, etc.), as well as the role of technological aids in it. Tables 1, 2 summarize the main findings of relevant empirical studies.

**Table 1:** Included empirical studies: Chinese-language publications.

Author	Title	Technology-assisted environment	Main findings (related to anxiety)
Watanabe (2020)	A study on Chinese learning anxiety of Japanese beginner-level learners in online classes	Online Chinese class	Anxiety levels were generally moderately high. The dimension of “Anxiety in Teacher-Student Classroom Communication” scored highest. Learning background (e.g., ethnicity, proficiency) was a primary influencing factor.
Guo (2021)	A Study on the anxiety in online spoken Chinese classes of international students at the primary stage	Online spoken Chinese class	Anxiety was prevalent among beginner learners. Individual factors (e.g., gender, proficiency) influenced anxiety levels. Students often employed positive coping strategies.
Chen C. (2021)	A study on the anxiety of junior and intermediate international students in Chinese online classes based on meta-cognitive strategies	Online Chinese class	Anxiety levels were moderate. Usage of meta-cognitive strategies showed a negative correlation with anxiety levels. Student individual factors and teacher instructional methods jointly influenced anxiety.
Xie and Luo (2021)	An analysis of the causes and adjustment strategies of anxiety in online Chinese language learning among international students	Online Chinese learning	Anxiety levels were higher compared to previous studies. Anxiety showed a negative correlation with HSK level and learning duration. High anxiety primarily stemmed from the course and exams themselves, rather than communication apprehension.
Sun W. R. (2022)	A study on the anxiety of online Chinese listening and speaking classes for high school students in Thailand	Online Chinese listening and speaking class	Moderate-to-high anxiety was prevalent. Anxiety was highest during the information processing stage. Listening and speaking materials were the most anxiety-inducing external factor.
Sun J. (2022)	A survey of Chinese learners' learning anxiety under network teaching environment	Network-based teaching environment	Anxiety levels were moderate. Affective deficiency anxiety (loneliness, lack of interaction) was most prominent. Age, HSK level, feelings of loneliness, and network factors significantly influenced anxiety.
Hong (2022)	A study on online writing anxiety and self-adjustment strategies of intermediate Chinese L2 learners	Online writing	Writing anxiety was at a moderate level. Anxiety was positively correlated with the frequency of self-regulated strategy use (learners with higher anxiety used strategies more frequently). Age and gender influenced specific anxiety dimensions.
Liu (2023)	A study on online Chinese language learning anxiety and coping ways of Pakistani students	Online Chinese learning	Anxiety levels were moderately high. Anxiety showed a negative correlation with the tendency to adopt positive coping strategies. Gender and self-rated language proficiency significantly affected anxiety.
Chen (2023)	A survey on online Chinese learning anxiety of primary level learners	Online Chinese learning	Anxiety levels were moderately high. Anxiety mainly stemmed from the external environment (e.g., limited teacher-student interaction, untimely feedback, network issues). Gender had an effect, but learning duration showed no significant impact.
Zhang et al. (2024)	The application of gamified virtual contexts in international Chinese language education—a utility perspective of discourse cognition and emotional motivation	Gamified virtual context	Gamified virtual contexts can significantly reduce language use anxiety, enhance willingness to communicate, and improve cognitive comprehension abilities.

**Table 2:** Included empirical studies: English-language publications.

Author	Title	Technology-assisted environment	Main findings (related to anxiety)
Wang et al. (2017)	Chinese language learners' anxiety toward chat partners in computer-mediated communication	Text-based Computer-Mediated Communication (CMC)	Chinese learners experienced significantly higher levels of Foreign Language Anxiety (FLA) when chatting with Native Speakers (NSs) compared to Non-Native Speakers (NNSs). The main mediating factors were language confidence and partner familiarity.
Xie et al. (2019b)	Using interactive virtual reality tools in an advanced Chinese language class: a case study	Virtual reality tools (Google cardboard/expeditions)	Using VR tools for contextualized presentations helped ease nervousness and anxiety during oral presentations.
Xie et al. (2019a)	Effects of using mobile-based virtual reality on Chinese L2 students' oral proficiency	Mobile-based Virtual Reality (VR: Google cardboard and expeditions)	Learners using VR tools for role-play tasks scored significantly higher on the content and vocabulary of their oral presentations than those using PowerPoint. VR reduced presentation anxiety by providing an immersive preparation environment and diverting audience attention.
Chen C. (2021)	The effects of online learning on alleviating students' Chinese language learning anxiety: a study in a Chinese University	Online learning platform	Online learning significantly reduced anxiety in listening, speaking, and writing, but increased anxiety in reading. Reading anxiety became the strongest predictor of general FLA in the online environment. Multiple regression indicated that reading anxiety explained more variance in general FLA for the online group than speaking anxiety did for the traditional group.
Xu et al. (2022)	The roles of motivation, anxiety and learning strategies in online Chinese learning among Thai learners of Chinese as a foreign language	Online learning	Anxiety emerged as the most stable negative predictor of self-rated CFL achievement in online learning.
Sandyaningrum and Kusumaningtyas, 2023	The effects of learning Mandarin online with a native lecturer to University students' anxiety	Online learning	Online learning with native lecturers caused student anxiety, manifesting as reduced learning conditions, self-efficacy, and learning effectiveness.
Srisinthon (2024)	Anxiety and engagement in the online classroom: a case study of Thai learners of Chinese as a foreign language	Online learning	Thai CFL learners' online learning anxiety was at a moderate level, primarily caused by fear of communication. Chinese proficiency affected anxiety, with low-proficiency learners having higher anxiety. Online learning anxiety was significantly correlated with learner engagement in the dimension of social presence.
Zhao and Wang (2024)	Domain-specific L2 grit, anxiety, boredom, and enjoyment in online Chinese learning	Online learning	In online Chinese learning, the Perseverance of Effort (POE) facet of L2 Grit significantly positively predicted learning enjoyment and negatively predicted anxiety. Both anxiety and enjoyment significantly predicted boredom.
Huang et al. (2024)	“How Anxious I am”: the effect of different online modalities on Chinese language beginners' classroom anxiety	Video conferencing (zoom) and virtual world (second life)	The effect of different online modalities on anxiety was complex, influenced by factors like gender, task-related language skills, and learners' immediate experience (e.g., engagement, task awareness). Reading anxiety decreased significantly in Zoom, while anxiety in speaking/listening decreased significantly in second life.
Puri et al. (2025)	The impact of mobile-based language learning on speaking and learning anxiety, engagement and achievement in Chinese language learning: the mediating role of cognitive load	Mobile-Based Language Learning (MBLL)	MBLL was significantly associated with lower speaking and learning anxiety. Cognitive load mediated this relationship, meaning MBLL reduces anxiety by lessening cognitive load.

**Figure 2:**
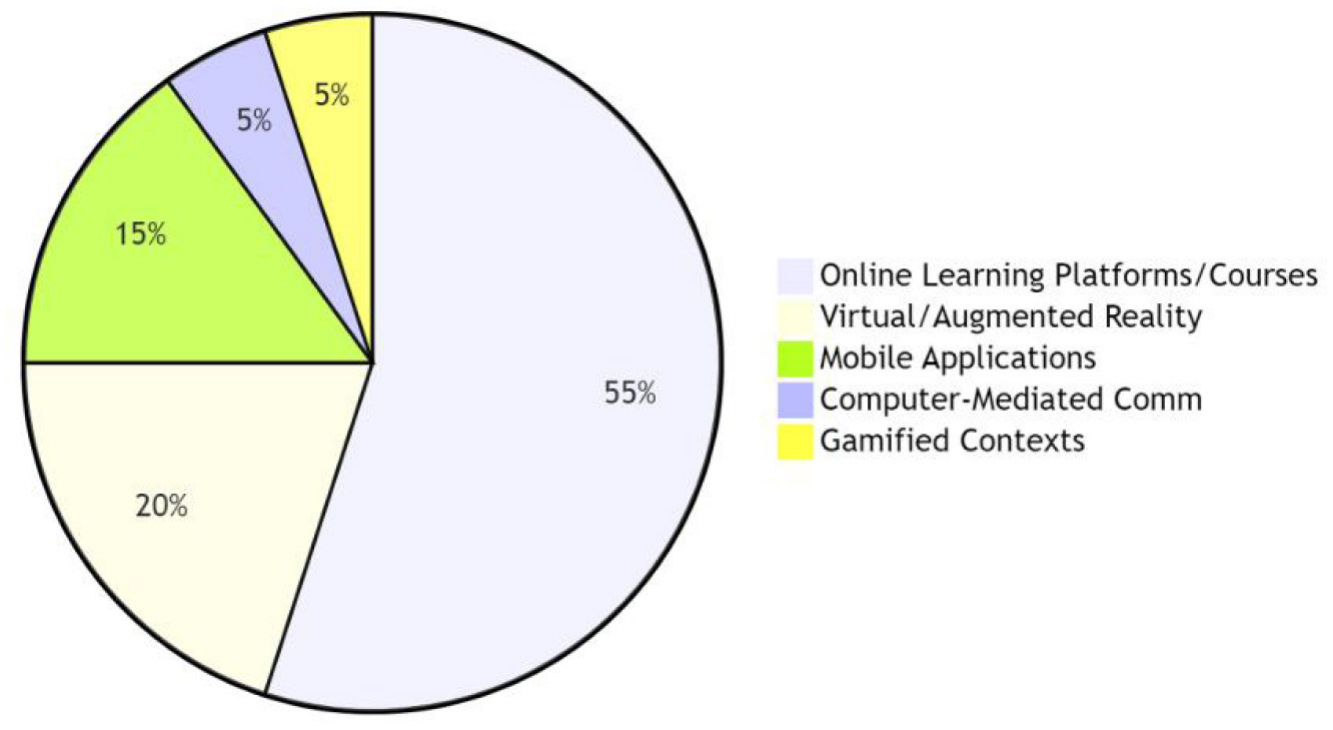
Distribution of technology-assisted environment types.

First of all, from the perspective of technology assisted environment (see Figure 2), online learning platforms (such as Zoom and various online course platforms) are mainstream research scenarios, which reflects the current trend of normalization of online education. At the same time, virtual reality/augmented reality (VR/AR) and mobile apps, as the intervention means of emerging technologies, have also attracted more attention, reflecting the emerging dynamics of technology development. However, the research on computer mediated communication (CMC) and gamified context is relatively few, and there is still room for further exploration.

**Figure 3:**
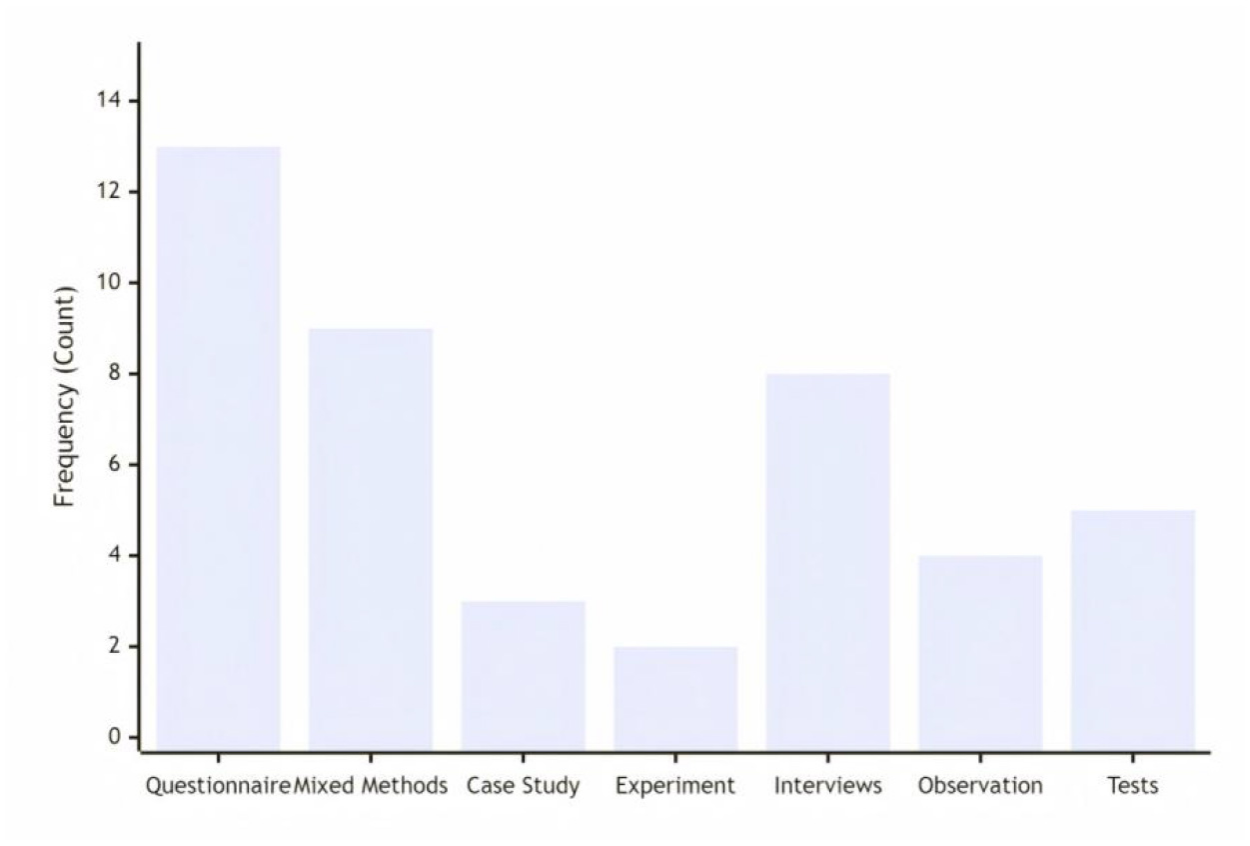
Distribution of research methodology types.

Quantitative research using questionnaires is dominant, as anxiety studies typically require large-scale data support. Mixed research methods (combined with questionnaires, interviews, observations, etc.) also account for a considerable proportion, which shows researchers' emphasis on in-depth exploration. Case studies and experimental studies provide strong evidence for in-depth understanding of individual differences and testing the effect of technical intervention (see Figure 3).

Existing empirical studies have shown that there are complex and diverse relationships between anxiety and Chinese second language learning. At the same time, as an important means of teaching intervention, technical aids have a significant impact on anxiety and related variables in many ways, and its effect mainly depends on the type of tools, the way of use and the learning situation. These findings provide an important empirical basis for further understanding the internal mechanism of Chinese second language acquisition and the design of effective teaching strategies.

## 3 Performance and influencing factors of Chinese second language anxiety in technology assisted environment

### 3.1 Multidimensional performance and skill differences of anxiety

The technology-assisted environment has reshaped the anxiety situation of Chinese second language learners. Research shows that online learners generally have moderately high levels of anxiety, and their manifestations exhibit significant multi-dimensional characteristics. On one hand, traditional classroom anxieties, such as communication anxiety and exam anxiety, still exist in the online environment and may even be amplified; on the other hand, the technological environment has given rise to new dimensions of anxiety, among which “emotional deprivation anxiety” becomes prominent due to physical isolation and insufficient social presence.

The technical operation anxiety in online Chinese language learning is increasingly prominent, involving multiple challenges such as network stability, platform operation, and adaptation to digital texts (Xie and Luo, 2021). The anxiety responses to language skills in the technical environment show significant differences. Listening comprehension often becomes an important source of anxiety for students due to the instantaneous and non-repetitive nature of information input in the online environment. Studies have shown that beginner learners frequently report that listening materials are too fast and content is difficult to understand, both of which directly contribute to listening anxiety. This issue is particularly pronounced in tasks with higher listening demands (Guo, 2021). In terms of oral expression, the “being watched” sensation during synchronous video interaction significantly increases students' psychological pressure. When learners express themselves in real time in front of the camera, they are prone to a strong sense of being evaluated, which further aggravates oral anxiety (Sun W. R., 2022). Writing learning also faces challenges in technical adaptation, such as difficulty in inputting Chinese characters and concerns about file saving, all of which may trigger writing anxiety (Hong, 2022). For intermediate-level learners, they not only need to adapt to the online writing environment but also overcome technical obstacles such as input method usage and format adjustment, which all increase the possibility of anxiety. Interestingly, reading anxiety may take on different forms in the online environment. Some research indicates that although online learning has a certain alleviating effect on anxiety in listening, speaking, and writing, differences in reading habits of digital texts, visual adaptation to screen reading, and other issues may also trigger or exacerbate reading anxiety (Chen C., 2021).

The specific manifestations of technical operation anxiety in various language skills have become an important psychological factor affecting online Chinese language learning. Future research can further focus on the construction of corresponding intervention strategies to enhance learners' learning experience and effectiveness in the technology-integrated environment.

The literature reveals a significant tension regarding the manifestation of communication anxiety, a core dimension of FLCAS, in online environments. On one hand, studies (e.g., Watanabe, 2020; Srisinthon, 2024) indicate that “Anxiety in Teacher-Student Classroom Communication” or fear of communication remains highly prevalent online, often cited as a primary or significant source of distress. This suggests a direct transference of traditional classroom anxieties to the digital space. On the other hand, other research (Chen D., 2021; Wang et al., 2017) presents a more nuanced picture, where the nature of the online partner (native vs. non-native speaker) or the specific task design (synchronous performance vs. asynchronous practice) critically mediates this anxiety. This contradiction points to a critical, underexamined variable: the specific affordances and constraints of the technological platform itself may reconfigure, rather than merely replicate, social evaluation dynamics. For instance, the “being watched” sensation on a synchronous video platform like Zoom may amplify the performative aspect of communication anxiety, while text-based CMC with delayed response could potentially mitigate the immediacy of social threat. Future syntheses must move beyond simply confirming the presence of communication anxiety online to dissecting how different technological mediation architectures reshape its intensity and qualitative experience.

The above discussion reveals the complexity of the effect of technology on anxiety. In order to understand these contradictory results more systematically, we need to examine several key regulatory variables. One is the technical mode. Synchronous video conferencing may aggravate performance anxiety, while asynchronous text communication or virtual environment may reduce social threats. Second is task design. Impromptu oral tasks are more likely to induce anxiety than prepared speeches, while game tasks can relieve pressure by reducing the cost of trial and error. Third is learners' level. Beginners' dual strangeness to technical operation and language itself may magnify anxiety, while middle and advanced learners are more affected by fear of evaluation. Finally, research design should be considered. Cross-sectional surveys can only capture static anxiety levels, while longitudinal or experimental designs can reveal the dynamic effects of technical intervention. Future research should clarify these regulatory factors in the design and explore the interaction between them, so as to more accurately explain the contradictory effect of technology.

### 3.2 Differentiated impact of individual factors

There is a deep intertwined relationship between learners' individual characteristics and anxiety experience. As a basic influencing factor, language proficiency has a significant effect on learners' anxiety. Primary learners often feel high anxiety because they are unable to understand and express effectively due to their immature language ability (Srisinthon, 2024). Intermediate learners, such as those who have reached HSK level 3–4, may encounter learning bottlenecks and face specific psychological difficulties, which will also cause anxiety (Sun J., 2022).

Self-cognition plays a key role in anxiety experience. Learners' underestimation of their language ability and uncertainty about the effectiveness of online learning will significantly aggravate their anxiety. This negative evaluation of self-ability often leads to learners' lack of self-confidence in the process of learning, and then increases anxiety. The influence of gender differences on anxiety experience in specific situations cannot be ignored. Female learners may be under greater pressure in terms of evaluation sensitivity and technology adaptability, which may result from excessive attention to others' evaluation and inadaptability to new technologies. For example, Pakistani female Chinese learners tend to show a higher level of anxiety in the face of technical operation and evaluation (Liu, 2023).

In addition, individual factors such as personality traits, motivation type, cultural background, and prior experience in a target-language country jointly shape differentiated anxiety responses. Introverted learners may be more likely to fall into an anxiety state because they are less likely to actively seek help and express themselves (Watanabe, 2020). However, learners with instrumental motivation, that is, those who learn Chinese mainly for practical purposes, may have higher anxiety levels due to excessive attention to learning outcomes (Xu et al., 2022).

In the future, when designing and implementing online Chinese teaching, we should fully consider these individual differences and adopt targeted teaching strategies to effectively alleviate learners' anxiety and improve their learning effect.

While the reviewed studies consistently identify individual factors (e.g., proficiency, gender, and motivation) as significant correlates of anxiety, the prevailing analytical mode tends to treat these as static, pre-existing learner attributes that predispose individuals to higher or lower anxiety in the technological environment. This perspective, while valuable, risks technological determinism by overlooking how the technological environment actively co-constructs these “individual” experiences. For example, the finding that female learners or beginners report higher anxiety should not be taken as a simple reflection of inherent traits. Instead, a critical analysis must ask: Does the design of the technology or the pedagogical approach enacted through it inadvertently create or exacerbate challenges that disproportionately affect these groups? A beginner's anxiety might be less about low proficiency itself more about the platform's lack of scaffolding or adjustable difficulty levels. Similarly, gender differences in anxiety could be linked to how online public performance or technical troubleshooting is socially framed within the learner's culture or the class dynamic. Therefore, future research should shift from merely cataloging which individual factors matter to investigating the interaction between learner characteristics and specific technological-pedagogical designs. This requires a design that can capture dynamic interactions, such as qualitative interviews, diary research and other methods, to deeply understand how different learners perceive and respond to specific technical characteristics, and how this perception changes over time. This would reveal whether technology is merely a neutral backdrop against which fixed differences play out, or an active agent that differentially modulates the anxiety landscape for diverse learners.

### 3.3 Interaction between teaching and technology environment

The interaction between teaching environment and technology design has a profound impact on the generation and evolution of learners' anxiety. The quality of interaction mode is a key variable affecting the level of anxiety. Although online communication with native speakers offers valuable language practice opportunities, it often leads to higher anxiety due to language proficiency gaps and social unfamiliarity (Wang et al., 2017). On the contrary, the lack or inefficiency of interaction between teachers and students and students directly leads to the lack of emotional support and the increase of learning frustration (Chen, 2023). This lack of emotional support further aggravates learners' anxiety and affects their learning enthusiasm and effect. Task design also plays an important role in the interaction between teaching and technology environment. Inappropriate task difficulty, insufficient preparation time and forced immediate performance may trigger learners' processing and output anxiety.

For example, in the online Chinese listening and speaking class, if the task design is too complex or the time arrangement is too tight, students often feel anxious because they cannot fully prepare (Sun W. R., 2022). Therefore, designing task difficulty reasonably, providing sufficient preparation time and avoiding forced immediate performance are effective ways to alleviate learners' anxiety.

The technological tool itself has duality, and whether its design is reasonable or not directly affects the anxiety level of learners. Improper technology design will become a source of anxiety, increase the cognitive burden and operation difficulty of learners, and cause anxiety. However, making good use of technical tools can become a pressure reducer. By reducing cognitive load, mobile learning provides learners with a more flexible and convenient way of learning, which indirectly alleviates the anxiety of learners, thus indirectly alleviating learning anxiety. In addition, virtual reality technology disperses learners' excessive attention to self-expression and reduces their anxiety through situational immersion (Xie et al., 2019a).

In order to effectively alleviate the anxiety of learners and improve their learning effect, teachers should pay attention to optimizing the interaction mode, reasonably designing the task difficulty, and making full use of the advantages of technical tools to create a positive and supportive learning environment for learners.

### 3.4 Complex interaction between emotion and cognition

Anxiety is not an isolated emotional phenomenon, but closely related to the cognitive process, which jointly affects learners' online learning experience. From the perspective of positive psychology, learners' psychological resilience, especially their efforts to adhere to this quality, can effectively buffer the negative effects of anxiety and promote the generation of positive emotional experience (Zhao and Wang, 2024). Specifically, when facing the challenges in online learning, learners with high psychological resilience are more able to maintain a positive attitude and turn anxiety into a driving force for progress, so as to improve the learning effect. Cognitive load theory further reveals the influence mechanism of technological environment on learners' anxiety level. In the technological environment, information overload is a common problem, which directly causes the anxiety reaction of learners. When the amount of information learners need to process exceeds the carrying capacity of their cognitive resources, cognitive overload will occur, which will lead to the rise of anxiety. Therefore, a good teaching design should focus on optimizing cognitive resources, help learners effectively manage cognitive load and reduce anxiety level by reasonably designing learning tasks and providing clear learning guidance. The development of meta-cognitive ability is particularly critical in anxiety management. Meta-cognition refers to learners' ability to recognize and regulate their cognitive process, including the use of strategies such as planning, monitoring and regulation. Research shows that learners who are good at using planning, monitoring, and adjustment strategies can show stronger adaptability and significantly lower anxiety level when facing the uncertainty of online learning (Chen D., 2021). These learners can make reasonable learning plans according to their own learning objectives and actual situation; In the process of learning, they can monitor their learning status in time, find problems, and adjust them in time; When facing difficulties and challenges, be able to actively seek solutions and maintain a positive learning attitude.

These findings all point to an important understanding: anxiety management in the technological environment needs to go beyond mere emotional comfort, but should go deep into the cognitive level and build a supportive learning ecology. This means that educators should not only pay attention to learners' emotional needs and provide emotional support; It is more necessary to help learners effectively manage cognitive load and enhance psychological resilience by optimizing teaching design and improving learners' meta-cognitive ability, so as to achieve a more efficient and pleasant learning experience in the technical environment. These cognitive and affective mechanisms not only explain the nature of anxiety in digital environments but also point toward potential leverage points for technological intervention, which we turn to next.

## 4 Strategies and empirical effects of technology intervention on Chinese second language anxiety

### 4.1 The interventional role of technology-enhanced environments in reducing anxiety

Immersive virtual environment provides an innovative path to alleviate anxiety through psychological transfer and situational embedding. The application of virtual reality technology shows that when learners act as virtual guides in VR scenes, their oral expression anxiety is significantly reduced. Its core mechanism is that the 360 degree immersion environment diverts the audience's attention from the speaker, providing a valuable psychological buffer (Xie et al., 2019b). This defocusing effect makes learners pay more attention to the task itself rather than self-expression.

Gamification design further expands the dimension of technical intervention. The study found that the anxiety level of primary Chinese learners who learned in the game virtual situation was significantly reduced after the intervention, and their willingness to communicate was improved (Zhang et al., 2024). Its utility mechanism is multiple. Game elements (such as progress visualization and achievement system) transform learning into a more attractive and secure exploration process by providing immediate positive feedback and controllable challenges; Virtual situation creates a low-risk language practice field, allowing learners to make bold attempts in an environment with low cost of making mistakes.

The design of interactive mode needs to be based on the refined consideration of technical characteristics. Comparative studies have found that different online environments have different effects on anxiety: learners who complete communication tasks in the three-dimensional virtual environment show lower overall anxiety than learners in the traditional video conference environment, especially in speaking and listening (Huang et al., 2024). This is because the avatars and situations provided by the virtual environment reduce the pressure to evaluate the real face and allow more flexible collaboration.

Even in the conventional platform, promoting structured social interaction through technical means can effectively alleviate the anxiety of emotional loss. These practices show that technology is not only a channel for information transmission, but also a catalyst for high-quality social interaction.

### 4.2 Technology-supported pathways for cognitive and meta-cognitive strategies

The intervention effect of mobile learning technology has been strongly supported by cognitive science theory. Well-designed mobile language learning applications can significantly reduce learners' oral anxiety and general learning anxiety, with cognitive load playing a key mediating role (Puri et al., 2025). This discovery has important teaching implications. The effectiveness of technological intervention depends not only on the richness of functions, but also on whether it conforms to the laws of human cognitive processing. When mobile applications can optimize the information processing process through simple navigation, timely support, personalized content push, etc., they can effectively reduce the external cognitive load, thus reducing the frustration and anxiety caused by it.

The combination of technical tools and strategy training provides an endogenous solution for anxiety management. Chen D. (2021) founds that in online Chinese learning, the frequency of meta-cognitive strategy use is significantly negatively correlated with the level of anxiety. Therefore, teachers can systematically integrate strategy training into the technical environment: guide students to use digital calendar and task management tools for learning planning (planning strategies); The learning process monitoring and self-assessment (monitoring strategy) are carried out by using the recording, broadcasting and playback function; Real time problem solving and path adjustment (adjustment strategy) using online resource library and help system. This training mode of technology empowerment strategy can enhance learners' sense of control over the online environment and fundamentally improve their adaptability.

### 4.3 Building a systematic technology-supported ecosystem

Effective technical intervention needs multi-level systematic support. For specific skill anxiety, targeted technical solutions should be adopted: to alleviate listening anxiety, multimodal audio and video resources with adjustable speech speed and optional subtitles can be provided; In order to reduce writing anxiety, online model article library, collaborative editing platform and intelligent feedback system can be built (Hong, 2022). In view of the general sense of technical unease, it is necessary to establish a perfect technical support system, including clear operation guidelines, timely technical assistance channels, and the recording and broadcasting backup of key teaching contents (Sandyaningrum and Kusumaningtyas, 2023). Furthermore, an ideal technology ecology should have integration and adaptability. It needs to organically combine immersive environment, mobile applications, interactive platform, and strategy training tools to form complementary technical solutions. At the same time, the ecosystem should be able to provide differentiated support paths according to learners' anxiety types, language levels, etc., such as automatically recommending learning resources or strategy guidance that match their current state according to learners' behavior data on the platform.

It is worth noting that the term “technology” in this review covers many levels, from hardware devices (such as VR head mounted display) to software platforms (such as zoom) and then to specific functions (such as asynchronous discussion and intelligent feedback). The effect is not single, but closely related to the specific implementation of these levels. The empirical studies reviewed, while demonstrating the potential of various technological interventions, collectively point to a crucial gap between technical feasibility and pedagogical sustainability in anxiety alleviation. A critical synthesis suggests that the effectiveness of any tool—be it VR for situational exposure, mobile apps for spaced repetition, or AI for personalized feedback—is not an inherent property of the technology itself. Instead, it is contingent upon its embeddedness within a coherent pedagogical design that addresses the core anxiety sources identified in Section 3. For instance, a gamified VR environment might successfully lower the cognitive load and immediate social threat for speaking practice (addressing performance anxiety), yet fail to mitigate a learner's underlying “fear of communication” if the activity is not followed by supportive, formative feedback that builds self-efficacy. Similarly, asynchronous tools that reduce evaluation apprehension must be carefully balanced with opportunities for developing communicative competence to prevent the reinforcement of avoidance strategies. Therefore, future intervention research should move beyond proving that a technology “can reduce anxiety” in a controlled setting. The priority should shift to investigating how these tools can be integrated into longitudinal, pedagogically-grounded learning sequences that not only provide temporary relief but also foster the psychological and strategic resources (e.g., enhanced self-regulation, positive attribution) needed for durable anxiety management. This necessitates study designs that evaluate not just pre-post anxiety scores, but also the mediating role of factors like growing familiarity with the tech environment, improved meta-cognitive awareness, and the quality of teacher/peer scaffolding within the digital space.

## 5 Conclusion

This mini review reveals the key findings in this field by combing the research on Chinese second language anxiety and its intervention in the technology assisted environment. The technological environment does not simply exacerbate or alleviate anxiety, but has a complex interaction with learners' individual characteristics, instructional design and language skills.

The study found that anxiety in the technology assisted environment showed obvious duality. On the one hand, the forms of communication fear and test anxiety in the traditional classroom are amplified in the online environment; On the other hand, the technological environment has given birth to new anxiety dimensions, such as emotional loss anxiety caused by physical isolation and technological operation anxiety caused by digital adaptation. The response of different language skills to the technical environment is significantly different. The transience of listening comprehension, the feeling of being watched during oral expression, the technical adaptability of writing and the habit of reading digital text together shape the differentiated anxiety experience. This complexity shows that the impact of technology on anxiety cannot be generalized, but needs to be analyzed in combination with specific skill areas.

In terms of intervention effect, research has confirmed the potential of technical means in alleviating anxiety, but its effectiveness highly depends on the specific implementation methods. Virtual reality and game design provide an effective solution to oral anxiety by creating a low-risk practice environment and a distraction mechanism; Mobile learning technology indirectly reduces learning pressure by optimizing cognitive load; The refined design of online interaction can alleviate the sense of social isolation. It is worth noting that the technological empowerment of meta-cognitive strategies shows its unique value. It enhances the learners' ability to cope with anxiety from the internal mechanism by enhancing the learners' sense of control over the learning process. These findings support the view that the fit with cognitive and emotional processes is key to the success of technical intervention (Golonka et al., 2014).

However, the current research has exhibits notable methodological limitations. A large number of studies are limited to the experimental design of small samples and short periods, relying too much on self-reported questionnaires, and lacking in-depth capture of the dynamic process of anxiety. At the theoretical level, the mediating mechanism of technology influencing anxiety has not been fully revealed, especially the path of how technology characteristics affect anxiety experience through variables such as cognitive load and self-efficacy needs to be further clarified. In addition, the existing studies focus on the negative effects of anxiety, and pay less attention to the complex relationship between anxiety and motivation. Some types of anxiety may promote learning, and the performance of this dimension in the technical environment is worth further discussion.

In general, the research on Chinese second language anxiety in the technology assisted environment is at a critical stage from phenomenon description to mechanism exploration. Future research needs to achieve breakthroughs in the preciseness of methods, theoretical depth and practical pertinence, so as to provide solid support for the construction of an efficient and humanized technology enhanced Chinese learning environment.

**Figure 4:**
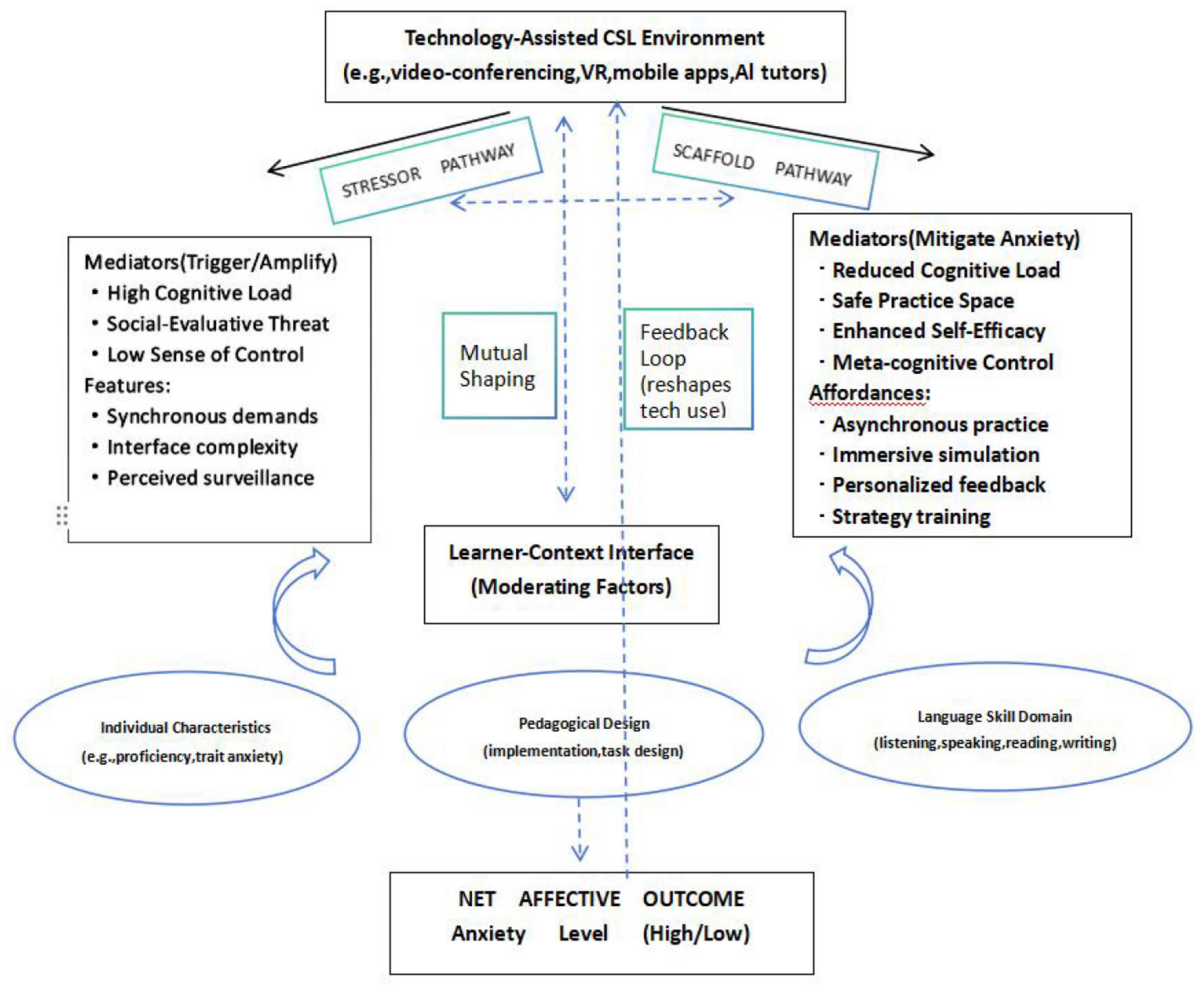
Technology-affect interaction framework.

To integrate these findings and guide future research beyond descriptive phenomenology, we propose a Technology-Affect Interaction Framework for conceptualizing anxiety in technology-assisted CSL learning (See Figure 4). This framework posits that learner anxiety in digital environments is not a direct result of technology *per se*, but the net outcome of a dynamic interaction between two core processes:

1. The Technology-as-Stressor Pathway: Certain technological features (e.g., synchronous performance demands, interface complexity, and perceived surveillance) can trigger or amplify anxiety by increasing cognitive load, heightening social-evaluative threat, and undermining the learner's sense of control.
2. The Technology-as-Scaffold Pathway: Conversely, other technological affordances (e.g., asynchronous practice, immersive simulation, personalized feedback, and strategy training tools) can mitigate anxiety by reducing extraneous cognitive load, offering a safe space for practice, and fostering self-efficacy and meta-cognitive control. The predominance of either pathway—and thus the ultimate affective outcome—is critically moderated by the learner-context interface: the learner's individual characteristics (e.g., proficiency, trait anxiety) interacting with the specific pedagogical design and the language skill domain in focus. This framework explains the documented duality of effects: the same technological environment (e.g., a video-conferencing platform) can activate different pathways depending on how its features are pedagogically implemented and filtered through individual and situational factors. Moving forward, this model directs research toward disambiguating these pathways, testing specific mediational chains (e.g., from technology feature X, through mediator Y, and to anxiety outcome Z), and designing interventions that systematically enhance the scaffolding pathway while mitigating the stressor pathway. Such a shift is essential for building the efficient and humanized technology-enhanced learning environments envisioned above.

## 6 Implications

Based on the analysis of existing literature, this study puts forward the following enlightenment from three aspects: theoretical construction, method innovation and practical application.

Theoretical construction needs to break through the traditional perspective that regards anxiety as a purely negative factor and establish a more dialectical theoretical framework. Future research can draw lessons from the research on the distinction between facilitating anxiety and inhibiting anxiety and their correlation with motivation (Luo et al., 2020), so as to further explore the dynamic transformation mechanism between them in the technological environment. At the same time, it is necessary to integrate cognitive load theory, social presence theory and self-regulated learning theory to build a comprehensive model that can explain how technical characteristics affect anxiety experience through multiple mediation paths. Special attention should be paid to the interaction between the uniqueness of Chinese language (such as Chinese character writing system, tone system) and the technical environment, so as to form an anxiety theory with the characteristics of Chinese second language learning.

Research methods should be diversified and deepened. First, expand the sample size and coverage of the study, and carry out long-term follow-up studies to avoid the result bias caused by novel effects. Secondly, using mixed research methods, such as the illustrative sequential design (Putri and Degeng, 2024), quantitative data are combined with qualitative data such as in-depth interviews and classroom observations to comprehensively capture the complex performance of anxiety. In terms of measurement tools, it is necessary to develop a new anxiety scale suitable for the technology assisted environment, which covers the dimensions of technology operation anxiety and digital social anxiety, and is supplemented by objective data such as physiological indicators, so as to improve the research validity.

Finally, the key to teaching practice is to build a learner centered technology enhanced learning ecology. Teachers and instructional designers should pay attention to the adjustment of intervention setting and duration, reasonably balance the learning arrangement between formal classroom and informal environment, and ensure the continuity and stability of technical intervention. According to the characteristics of different language skills, differentiated support strategies are designed, such as using VR technology to alleviate oral anxiety, providing adjustable listening materials to reduce listening pressure, and establishing online writing communities to enhance writing confidence. Meta-cognitive strategy training is integrated into the process of using technical tools to cultivate learners' ability to plan, monitor and adjust learning, and fundamentally improve their adaptability and anti-anxiety ability in the technical environment. Correctly guide learners to understand the pressure in the technology learning environment, and transform moderate anxiety into learning motivation through goal setting and positive feedback, so as to avoid the negative effects of excessive anxiety. For technology developers, they should pay attention to user experience design, reduce the threshold of technology operation, and provide personalized learning support and emotional feedback. Technology design should serve the goal of teaching method rather than the opposite, and avoid technology itself becoming a new source of anxiety.

Future research can be carried out from the following aspects: first, to explore the mediating mechanism of technology affecting anxiety and clarify its internal path; The second is to carry out cross-cultural and cross-age comparative research to reveal the differences and similarities between different groups; The third is to explore the application potential of artificial intelligence technology in personalized anxiety intervention and promote practical innovation; Fourth, on this basis, it is committed to building a comprehensive Chinese learning support system integrating technical support, teaching design, and emotional care.

## References

[B1] Burston J. (2015). Twenty years of MALL project implementation: a meta-analysis of learning outcomes. ReCALL 27, 4–20. doi: 10.1017/S0958344014000159

[B2] Chen C. (2021). The effects of online learning on alleviating students' Chinese language learning anxiety: a study in a Chinese university. Front. Educ. Technol. 4, 85–107. doi: 10.22158/fet.v4n2p85

[B3] Chen D. (2021). A study on the anxiety of junior and intermediate international students in Chinese online classes based on meta-cognitive strategies (Master's thesis). Shanghai Normal University, Shanghai, China.

[B4] Chen S. (2023). A survey on online Chinese learning anxiety of primary level learners (Master's thesis). Yunnan Normal University, Kunming, China.

[B5] Dong L. Jamal Mohammed S. Ahmed Abdel-Al Ibrahim K. Rezai A. (2022). Fostering EFL learners' motivation, anxiety, and self-efficacy through computer-assisted language learning- and mobile-assisted language learning-based instructions. Front. Psychol. 13:899557. doi: 10.3389/fpsyg.2022.899557 36033068 PMC9416476

[B6] Golonka E. M. Bowles A. R. Frank V. M. Richardson D. L. Freynik S. (2014). Technologies for foreign language learning: a review of technology types and their effectiveness. Comput. Assist. Lang. Learn. 27, 70–105. doi: 10.1080/09588221.2012.700315

[B7] Guo T. F. (2021). A study on the anxiety in online spoken Chinese class of international students at the primary stage (Master's thesis). Zhejiang University of Science and Technology, Hangzhou, China.

[B8] Hong Y. (2022). A study on online writing anxiety and self adjustment strategies of intermediate Chinese learners as second language (Master's thesis). Shanghai International Studies University, Shanghai, China.

[B9] Horwitz E. K. Horwitz M. B. Cope J. (1986). Foreign language classroom anxiety. Mod. Lang. J. 70, 125–132. doi: 10.1111/j.1540-4781.1986.tb05256.x

[B10] Huang H. Grant S. Yan J. (2024). ‘How anxious I am': the effect of different online modalities on Chinese language beginners' classroom anxiety. Lang. Learn. J. 52, 539–555. doi: 10.1080/09571736.2024.2365375

[B11] Kukulska-Hulme A. Viberg O. (2018). Mobile collaborative language learning: state of the art. Br. J. Educ. Technol. 49, 207–218. doi: 10.1111/bjet.12580

[B12] Lan X. (2025). A systematic review of anxiety, motivation, and strategy in learning Chinese as a second language from 2008 to 2022. Chin. J. Appl. Linguist. 48, 325–351. doi: 10.1515/CJAL-2025-0209

[B13] Liu J. J. (2023). A study on online Chinese language learning anxiety and coping ways of Pakistani students: taking Hebei University as an example (Master's thesis). Hebei University, Baoding, China.

[B14] Luo Z. Subramaniam G. O'Steen B. (2020). Will anxiety boost motivation? The relationship between anxiety and motivation in foreign language learning. Malays. J. ELT Res. 17, 53–71.

[B15] Lyu B. Qi X. (2020). A review of research on technology-assisted teaching and learning of Chinese as a second or foreign language from 2008 to 2018. Front. Educ. China 15, 142–163. doi: 10.1007/s11516-020-0006-8

[B16] Puri B. Mushtaque I. Fang S. Chenhe G. Younas A. (2025). The impact of mobile-based language learning on speaking and learning anxiety, engagement and achievement in Chinese language learning: the mediating role of cognitive load. Acta Psychol. 259:105400. doi: 10.1016/j.actpsy.2025.105400 40795447

[B17] Putri N. A. Degeng P. D. D. (2024). Utilizing Mobile-Assisted Language Learning (MALL) to alleviate speaking anxiety among EFL students. Engl. Rev. J. Engl. Educ. 12, 125–136. doi: 10.25134/erjee.v12i1.9352

[B18] Qian X. (1999). Foreign students' anxiety in learning Chinese. Lang. Teach. Linguist. Stud., 144–154.

[B19] Qiao P. Yuan Z. Li S. (2025). The impact of anxiety on Chinese as a second language achievement: a meta-analytic perspective. Front. Psychol. 16:1620275. doi: 10.3389/fpsyg.2025.1620275 41030346 PMC12477158

[B20] Sandyaningrum J. N. Kusumaningtyas D. P. S. (2023). The effects of learning Mandarin online with a native lecturer to university students' anxiety. Mandarinable J. Chin. Stud. 1, 121–129. doi: 10.20961/mandarinable.v2i2.947

[B21] Srisinthon P. (2024). Anxiety and engagement in the online classroom: a case study of Thai learners of Chinese as a foreign language. Knowl. Manag. Elearn. 16, 716–735. doi: 10.34105/j.kmel.2024.16.033

[B22] Stockwell G. (2013). “Technology and motivation in English-language teaching and learning,” in International Perspectives on Motivation: Language Learning and Professional Challenges, ed. E. Ushioda (London: Palgrave Macmillan UK), 156–175. doi: 10.1057/9781137000873_9

[B23] Sun J. (2022). A survey of Chinese learners' learning anxiety under network teaching environment (Master's thesis). Dalian University of Foreign Languages, Dalian, China.

[B24] Sun W. R. (2022). A study on the anxiety of online Chinese listening and speaking classes for high school students in Thailand (Master's thesis). Lanzhou Jiaotong University, Lanzhou, China.

[B25] Wang Y. Crooks S. M. Borst S. (2017). Chinese language learners' anxiety toward chat partners in computer-mediated communication. Chin. Second Lang. 52, 127–147. doi: 10.1075/csl.52.2.02wan

[B26] Watanabe A. (2020). Beginner level Japanese students online language learning anxiety (Master's thesis). Beijing Foreign Studies University, Beijing, China.

[B27] Xie Y. Chen Y. Ryder L. H. (2019a). Effects of using mobile-based virtual reality on Chinese L2 students' oral proficiency. Comput. Assist. Lang. Learn. 34, 1–21. doi: 10.1080/09588221.2019.1604551

[B28] Xie Y. Ryder L. Chen Y. (2019b). Using interactive virtual reality tools in an advanced Chinese language class: a case study. TechTrends 63, 251–259. doi: 10.1007/s11528-019-00389-z

[B29] Xie Y. J. Luo T. (2021). An analysis of the causes and adjustment strategies of anxiety in online Chinese language learning among international students. J. Hunan Mass Media Vocat. Tech. Coll. 21, 77–81. doi: 10.16261/j.cnki.cn43-1370/z.2021.02.019

[B30] Xu W. Zhang H. Sukjairungwattana P. Wang T. (2022). The roles of motivation, anxiety and learning strategies in online Chinese learning among Thai learners of Chinese as a foreign language. Front. Psychol. 13:962492. doi: 10.3389/fpsyg.2022.962492 36051198 PMC9426342

[B31] Yao S. Zhang D. Shen Q. (2022). Research on anxiety of learning Chinese as a second or foreign language in and outside Mainland China: a systematic review of the literature 1999–2020. Front. Psychol. 13:843858. doi: 10.3389/fpsyg.2022.843858 35360624 PMC8960163

[B32] Zhang L. Fang L. Shang J. J. (2024). The application of gamified virtual contexts in international Chinese language education: a utility perspective of discourse cognition and emotional motivation. Eeduc. Res. 45, 121–128.

[B33] Zhao X. Wang D. (2024). Domain-specific L2 grit, anxiety, boredom, and enjoyment in online Chinese learning. Asia-Pac. Educ. Res. 33, 783–794. doi: 10.1007/s40299-023-00777-3

